# Innominate artery aneurysm in behcet disease; report of one case

**DOI:** 10.1016/j.ijscr.2024.109314

**Published:** 2024-02-02

**Authors:** Mohammad Mozafar, Dorsa Najari, Meisam Refaei, Mohsen Sheikhzadeh, Mohammad Moein Mirhosseini

**Affiliations:** Division of Vascular and Endovascular Surgery, Department of Surgery, Shohada-Tajrish Medical Center, Shahid Beheshti University of Medical Sciences, Tehran, Iran

**Keywords:** Innominate artery, Aneurysm, Behcet disease, Case report, Endovascular

## Abstract

**Introduction:**

Behçet disease (BD) is a multisystemic recurrent inflammatory disorder that was originally described as a triad of oral and genital ulcerations with uveitis (Behcet, 1937 [1]). Arterial involvement is the most common cause of mortality in patients with BD. Aneurysms are common among the arterial lesions and affect various arteries, but mostly the abdominal aorta. Vascular lesions are encountered in 7 %–29 % of patients, gravely affecting the course of the disease. Extracranial carotid aneurysms due to Behçet's disease are extremely rare (Bouarhroum et al. (2006) [2]).

**Case presentation:**

Herein, we present a 19 year old man presented with hoarsness due to pressure effect to our outpatient clinic.

**Clinical discussion:**

Due to findings in the computed angiography, he underwent surgery twice.A 100*8 COVERA-covered stent was deployed at the bifurcation of the brachiocephalic artery. Then a 40*13.5 FLUENCY stent with a 2 cm overlap from the previous stent was deployed.

**Conclusion:**

Further investigations regarding endovascular approach for this rare disease is recommended.

## Highlights

• Arterial involvement is the most common cause of mortality in patients with Behcet disease. However; extracranial carotid aneurysms due to Behçet's disease are extremely rare.
• Surgical and endovascular treatment options should be evaluated without delay before a possibly irreversible complication occurs.
• To prevent side effects, nowadays, endovascular stent-graft repair has been a modality of choice for some clinicians.

## 1 Background

Since Behcet described the triad of oral ulcers, genital ulcers, and ocular lesions in 1937, many series have reported vascular manifestations in the course of Behcet's disease (BD) [1]. Vascular manifestations have mainly involved the venous system, but arterial lesions have also been reported. The pathogenesis of aneurysmal degeneration is thought to be vasculitis, resulting in obliterative endarteritis of the vasa vasorum supplying the medium-sized and large-sized vessels [3]. The most commonly affected arteries are the abdominal aorta, the femoral and pulmonary arteries, and the subclavian, popliteal, and common carotid arteries [4]. However, every major artery can be affected, including the coronary, axillary, brachial, radial, ulnar, iliac, and tibial arteries. In general, extracranial carotid artery aneurysms (ECAAs) are extremely rare, accounting for less than 2 % of carotid surgery [2].

Cardiovascular involvement can include both arteries and veins, with lesions ranging from arterial occlusion and aneurysms to superficial thrombophlebitis and occlusion of the superior or inferior vena cava. Most vascular deaths are related to the rupture of aneurysms. Clinical manifestations of Behcet's disease are mostly due to systemic vasculitis. Compared to all other systemic vasculitis, Behcet's disease is notable for its potential to affect blood vessels of all sizes (large, medium, and small) on both the venous and arterial sides of the vasculature. Deep venous thrombosis and thrombophlebitis are the most frequent vascular manifestations, followed by an arterial aneurysm and obstruction [5]. Arterial aneurysms mostly occur in the abdominal aorta and pulmonary arteries [2]. According to the recent literature, less than 12 cases of extracranial carotid artery aneurysm have been reported [2]. Moreover, innominate artery aneurysms seem rare, and only one case has been reported in 1987 in French literature and one ruptured case in an elderly man in Japanese literature [3,4]. Above all, vascular complications are the most important predictors of mortality and morbidity in Behcet's disease, and arterial aneurysms have been the leading cause of death because they are known to expand rapidly and result in fatal rupture.Appropriate medical treatment should be constituted as far as possible, and surgical and endovascular treatment options should be evaluated without delay before a possibly irreversible complication occurs.

The work in this case presentation has been reported in line with the SCARE criteria, and the patient provided informed consent [6].

## 2 Our case

A 19-year-old man with a history of oral ulcers and Behcet disease that was confirmed with a pathology test developed a mass on the right side of the neck. He complained of hoarseness. He possessed no cardiovascular risk factors. There was no history of trauma, surgery, or irradiation on his chest. The neurologic examination was normal. He was treated with Azathioprine 100 mg daily and Prednisolone 20 mg daily, but it seemed that he did not have complete compliance with therapy; however, according to the latest rheumatological reports prior to his admission to our facility, his BD was under control and no hypercoagulable symptoms were observed.

Laboratory data right after admission were all normal. After admitting the patient, with all his past history in mind, we requested a contrast-enhanced chest computed tomography angiography (CTA) with suspicion of a vascular pathology causing pressure effects on the recurrent laryngeal nerves.

**Figs. 1 and 2:**
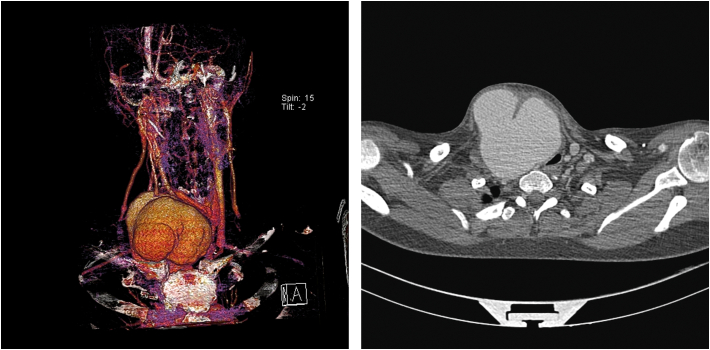
Preoperative contrast-enhanced computed tomography angiogram (CTA): the pseudoaneurysm of the innominate artery causing pressure effect on the trachea.

The computed tomography angiography (Figs. 1 and 2) showed a saccular aneurysm at the bifurcation of the right brachiocephalic artery (97*95*107 mm). The aneurysm compressed the internal jugular vein and caused shifting of the trachea. This compression to the adjacent parts like vocal cords, caused hoarseness.

**Fig. 3:**
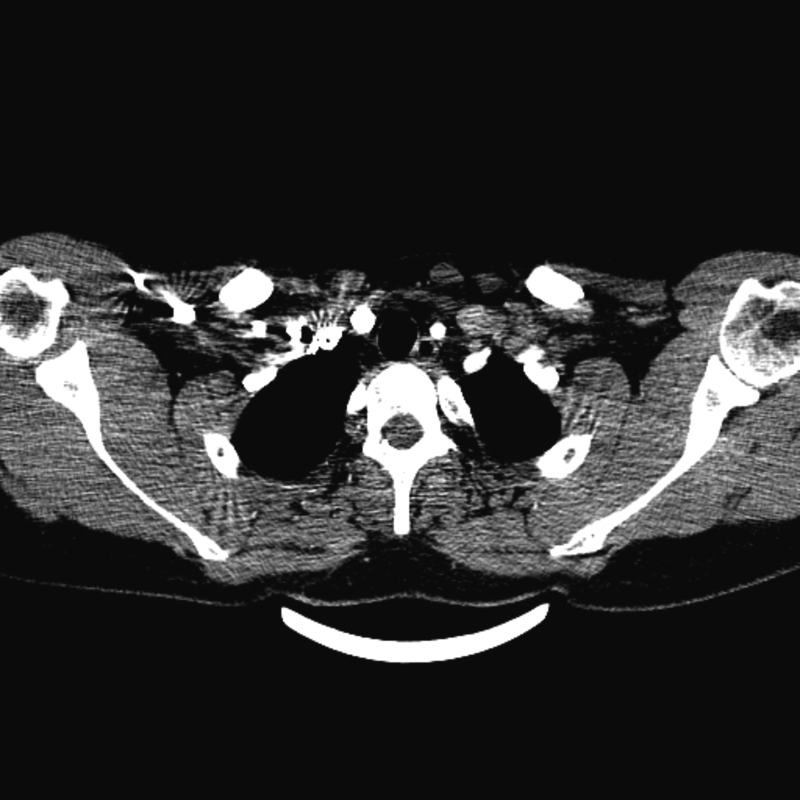
Post-operative contrast-enhanced computed tomography angiogram (CTA).

Preoperatively, the patient received methylprednisolone to decrease postoperative complications. In the OR, access was from the femoral artery, and angiography was performed with a 5Fr sheath, then a 12Fr (85 cm) sheath was prepared, and selective angiography was performed. A 100*8 COVERA-covered stent was deployed at the bifurcation of the brachiocephalic artery. Then a 40*13.5 FLUENCY stent with a 2 cm overlap from the previous stent was deployed. Control angiography showed endoleak, so from the brachial artery with the 5Fr sheath and with the use of a vertebral catheter at the endoleak location, three coils (5, 6, and 8) were deployed. Two weeks post operation, in order to removing the aneurysm sac completely, we operated on him again and we cut open the sac, later, the control angiography showed that the endoleak was stopped and the pressure effect of the pseudoaneurysm was removed. (Fig. 3).

The postoperative course was uneventful, and later, the patient was transferred to the rheumatology ward. The hoarseness of the patient resolved through the hospital course. In the follow-up sessions during the next year, no sign of recurrence was shown and he is prescribed Tab ASA 80 daily alongside with his previous medications.

## 3 Discussion

Behcet disease is a multisystemic disorder characterized by oral ulceration plus at least two of the following: recurrent genital ulcerations, eye lesions (such as uveitis), skin lesions (such as erythema nodosum or folliculitis), and a positive pathergy test [7]. Vascular involvement appears in 7 % to 29 % of patients with BD and gravely affects the course of the disease. Vascular lesions are most likely to involve the venous system; however, arterial lesions are associated with a greater risk [8]. The pathogenesis of aneurysmal degeneration is thought to be vasculitis resulting in obliterative endarteritis of the vasa vasorum supplying the medium-sized and large-sized vessels [9]. Regarding to the risk of rupture and poor responsiveness to medical therapy, the surgical treatment should be considered such as open or endovascular treatment. Additionally, adjuvant immunotherapy, with or without high doses of corticosteroids, should be used to control the formation of new aneurysms and to minimize the risk of graft occlusion [7]. Surgical intervention in the active inflammatory phase is also problematic because of the potential risks of anastomotic pseudoaneurysm formation and graft occlusion,and most authors recommended avoiding surgery during the acute inflammatory phase of the disease [10]. It is reported that during this phase, even a diagnostic angiogram is associated with an increased risk of false aneurysm formation at the puncture site [11]. Various surgical approaches have been practiced to manage innominate artery aneurysms, such as endovascular surgery and partial or full median sternotomy with or without anterior neck and supraclavicular fossa dissection [12].

Nowadays, endovascular stent-graft repair has been a modality of choice for some clinicians. Moreover, Park et al. reported an acceptable result of 7 cases with aortic or arterial aneurysms in BD treated with stent-graft insertions [13].

Postoperative corticosteroid therapy and systemic immunosuppression with azathioprine, chlorambucil, or cyclophosphamide have been suggested as effective prophylactic agents for arterial relapse, including aneurysm formation at anastomotic sites and other arteries. Moreover, as mentioned in recent articles, Vascular surgery is challenging in BD patients and the mortality rate is high, mainly because of anastomotic aneurysm relapses and graft thrombosis [14].

## 4 Conclusion

Most patient with carotid artery aneurysm & pseudoaneurysm were treated by open surgical procedure and further investigation such as our case report with long-term follow-up is recommended to determine the efficacy of endovascular approach, cause although the effectiveness of endovascular stent-graft repair to avoid these consequences has just been described, experience is still limited due to the disease's rarity.

## Consent

Written informed consent was obtained from the patient for publication and any accompanying images. A copy of the written consent is available for review by the Editor-in-Chief of this journal on request.

## Ethical approval

It was not required in this study, because we did not proceed with a new method.

## Funding

No sources of funding is available.

## Author contribution

Dorsa Najari: Writing the paper.

Meisam Refaei: Data collection, Revising the paper.

Mohammad Mozafar:Revising the paper.

Mohsen Sheikhzadeh:Data curation, Revising the paper.

Mohammadmoein Mirhosseini: Study concept,writing the paper.

## Guarantor

Mohammad Mozafar.

## Conflict of interest statement

There is no confliction of interest.
